# HIV status, breastfeeding modality at 5 months and postpartum maternal weight changes over 24 months in rural South Africa

**DOI:** 10.1111/tmi.12320

**Published:** 2014-04-11

**Authors:** Terusha Chetty, Rosalind J Carter, Ruth M Bland, Marie-Louise Newell

**Affiliations:** 1 Africa Centre for Health and Population Studies, University of KwaZulu-NatalSomkhele, South Africa; 2 Department of Epidemiology, Mailman School of Public Health, Columbia UniversityNew York, NY, USA; 3 Royal Hospital for Sick Children, University of GlasgowGlasgow, UK; 4 Faculty of Medicine, University of SouthamptonSouthampton, UK

**Keywords:** HIV infection, body weight change, breastfeeding, postpartum

## Abstract

**Objective:**

To determine the effect of infant feeding practices on postpartum weight change among HIV-infected and -uninfected women in South Africa.

**Methods:**

In a non-randomised intervention cohort study of antiretroviral therapy-naïve women in South Africa, infants were classified as exclusive (EBF), mixed (MF) or non-breastfed (NBF) at each visit. We analysed infant feeding cumulatively from birth to 5 months using 24-hour feeding history (collected weekly for each of the preceding 7 days). Using generalised estimating equation mixed models, allowing for repeated measures, we compared postpartum weight change (kg) from the first maternal postpartum weight within the first 6 weeks (baseline weight) to each subsequent visit through 24 months among 2340 HIV-infected and -uninfected women with live births and at least two postpartum weight measurements.

**Results:**

HIV-infected (−0.2 kg CI: −1.7 to 1.3 kg; *P *=* *0.81) and -uninfected women (−0.5 kg; 95% CI: −2.1 to 1.2 kg; *P *=* *0.58) had marginal non-significant weight loss from baseline to 24 months postpartum. Adjusting for HIV status, socio-demographic, pregnancy-related and infant factors, 5-month feeding modality was not significantly associated with postpartum weight change: weight change by 24 months postpartum, compared to the change in the reference EBF group, was 0.03 kg in NBF (95% CI: −2.5 to +2.5 kg; *P *=* *0.90) and 0.1 kg in MF (95% CI: −3.0 to +3.2 kg; *P *=* *0.78).

**Conclusion:**

HIV-infected and -uninfected women experienced similar weight loss over 24 months. Weight change postpartum was not associated with 5-month breastfeeding modality among HIV-infected and -uninfected women.

## Introduction

HIV infection can be associated with wasting, progressive loss of fat-free and fat mass, amplifying morbidity and mortality (Macallan 1999). Weight loss in HIV-infected individuals is generally multicausal (Mulligan *et al*. 1997); energy requirements may need to increase by 10% in asymptomatic adults to maintain body weight (Kosmiski 2011) and 20%–30% in symptomatic adults (2003a).

Lactating women experience gradual weight loss during the first 6 months, varying within and across populations (Butte & Hopkinson 1998); some undernourished populations gain weight (Hartmann *et al*. 1998; Winkvist *et al*. 2003). Ideally, nutrients stored antenatally, including fat stored energy, should be available during lactation (Butte & King 2005).

Little is known about HIV and maternal nutritional status. In developing countries, HIV-infected women may be vulnerable to nutrient deficiencies from inadequate dietary intake, nutritional demands imposed by HIV and other infections, and pregnancy and lactation nutritional requirements (Nduati *et al*. 2001). These demands may have harmful effects on maternal health, including postpartum weight loss, possibly accelerating HIV disease progression (Kotler *et al*. 1989; Palenicek *et al*. 1995; Nduati *et al*. 2001). In Kenya, breastfeeding was associated with weight loss (Nduati *et al*. 2001), but with weight gain in Zambia (Murnane *et al*. 2010). Other studies of postpartum weight change have observed weight loss (Papathakis *et al*. 2006), equivalent (Koyanagi *et al*. 2011) and less weight gain (Ladner *et al*. 1998) in HIV-infected women relative to HIV-uninfected women.

Weight change during lactation may have implications for maternal and infant health (Nduati *et al*. 2001). This study aims to assess postpartum weight change through 24 months in HIV-infected and -uninfected women in South Africa and evaluate the effect of infant feeding practice at 5 months (World Health Organization 2003), particularly among antiretroviral therapy (ART)-naïve HIV-infected women.

## Methods

This study analysed data collected from the Vertical Transmission Study (VTS), a non-randomised intervention cohort study, in KwaZulu-Natal, South Africa (Coovadia *et al*. 2007; Rollins *et al*. 2008; Bland *et al*. 2010). The VTS was designed to investigate the effects of infant feeding practices of HIV-infected women on HIV transmission and infant survival (Rollins *et al*. 2008); 3445 women were enrolled during pregnancy between 2001 and 2004 from rural and peri-urban clinics (Bland *et al*. 2010). Eligibility for the VTS included age ≥16 years, minimum of 3 months of residence within the study area postpartum and written informed consent (Bland *et al*. 2010).

Lay counsellors counselled women at clinics and supported their feeding choice based on WHO 2000 and 2003 infant feeding recommendations (WHO/UNICEF/UNAIDS 2000; WHO/UNICEF/UNAIDS/UNFPA 2003; World Health Organization 2003; Bland *et al*. 2007). According to this WHO recommendation, ‘infants should be exclusively breastfed for the first 6 months of life (World Health Organization 2003)’. Furthermore, when infants were 5 months of age, breastfeeding mothers were counselled to stop breastfeeding (at 6 months) infants who were confirmed HIV -uninfected; mothers were to continue breastfeeding infants who were HIV -infected (Rollins *et al*. 2008). Antenatally, lay counsellors visited enrolled women at home, to discuss study logistics and collect socio-demographic and pregnancy data.

HIV-infected women and their infants received single-dose nevirapine according to the KwaZulu-Natal prevention of mother-to-child transmission (PMTCT) policy (KwaZulu-Natal Provincial Department of Health 2003). No VTS mothers received ART during or the year after pregnancy as treatment was unavailable in the government health services before late 2004 (Houlihan *et al*. 2009; Bland *et al*. 2010). Women who delivered outside of the VTS facility were encouraged to attend the clinic as soon as feasible; women were also encouraged to attend the clinic around week six postpartum, but could attend earlier if more convenient. From 6 weeks postpartum, study nurses assessed women monthly for 9 months, then three-monthly to 24 months. At each clinic visit, medical history was documented, and women had physical and anthropometric examinations [weight, height, mid-upper arm circumference (MUAC)] using standardized equipment (Bland *et al*. 2010).

Women's height was not included in the original VTS protocol, but was added after December 2001, thus 47.3% of participants had height measurements; height was measured without shoes to the nearest centimetre using a stadiometer. Weight was measured to the nearest 100 g using a calibrated electronic digital scale (Scales 2000, Durban, South Africa) (Bland *et al*. 2010). Two separate weight, height and MUAC measurements were taken at every clinic visit.

Within 72 h postpartum, breastfeeding counsellors visited women at home, regardless of feeding intentions (Bland *et al*. 2010). Women choosing breastfeeding were supported with home visits from breastfeeding counsellors three times in the first 2 weeks postpartum, then twice weekly until 6 months (Bland *et al*. 2008). Clinic staff and an infant feeding specialist supported women choosing to formula-feed (Coovadia *et al*. 2007); the PMTCT programme provided free infant formula for HIV-infected women as appropriate from the end of 2002 (Bland *et al*. 2010). Field monitors conducted weekly home visits till 9 months for all women regardless of feeding practice (Bland *et al*. 2010), recording data on feeding, liquids, medications, morbidity, hospitalizations and health facility attendances for each day of the previous week (Bland *et al*. 2010). Women kept infant food intake and morbidity diaries to corroborate the field monitor interviews (Bland *et al*. 2010).

Antenatally, blood for CD4^+^ counts (September 2001–March 2003; FACScan; Becton, Dickinson and Company, New Jersey, USA; then Epics XL; Beckman Coulter Inc, California, USA) was collected (Dieye *et al*. 2005). Maternal venous blood samples were also taken at 6 months postpartum for CD4^+^ count and HIV RNA viral load (Bland *et al*. 2010).

All women signed informed consent; the University of KwaZulu-Natal Biomedical Research Ethics Committee (T050/01) approved the study (Rollins *et al*. 2008).

### Study population

**Figure 1:**
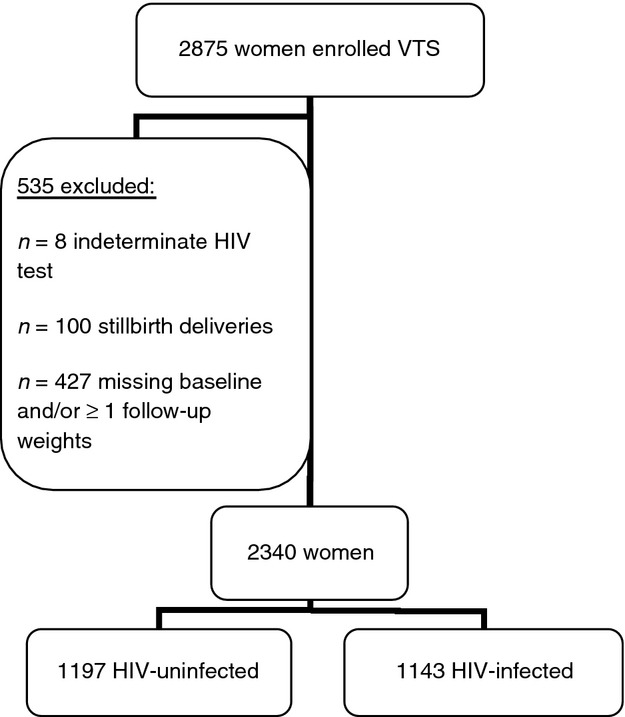
VTS sample flow chart.

We included HIV-infected and -uninfected women with up to 24 months of follow-up postpartum. Exclusions were women with indeterminate antenatal HIV test results (*n *=* *8), stillbirths (*n *=* *100) or missing baseline and at least one follow-up weight (*n *=* *427) as weight changes could not be assessed (Figure 1). Follow-up was censored at 24 months. None of the women included in this study had subsequent pregnancies in the 24 months after delivery of the first infant.

### Statistical analyses

Differences between HIV-infected and -uninfected subjects were analysed using a chi-square or Fisher's exact test for categorical variables and Student's *t-*test for continuous variables. As women in the study had two weight measurements at each clinic visit, the mean weight for each woman at each visit was calculated. BMI was calculated for individuals with heights. The baseline weight was the first maternal weight within 6 weeks postpartum (including weight measurements from delivery). The dependent variable was the mean weight change (kg) from the baseline weight in HIV-infected and -uninfected women at each study visit. Low MUAC was defined as <23 cm (fifth percentile of the United States National Health and Nutrition Examination Survey) (Center for Disease Control 2008).

We analysed feeding by classifying feeding on each day of life, then cumulative patterns from birth using 24-hour feeding histories according to WHO definitions: (i) Exclusive breastfeeding (EBF) if breastfeeding only with no food or drink, even water, with oral rehydration solution, drops and syrups (vitamins, minerals and medicines) allowed; (ii) non-breastfeeding (NBF), if replacement feeding exclusively or replacement feeding and other solids were reported; and (iii) mixed feeding (MF), if feeding with breast milk and formula milk, or breast milk and solids. Women were classified as predominant breastfeeders or MF at the time of introduction of water, or other liquids or solids, respectively. Predominant breastfeeders were combined with EBF as we assumed the mean weight change from baseline in these groups would be similar. Infant feeding data were assessed cross-sectionally at 5 months as any effect of feeding would be expected to evident by then and due to the decreasing sample sizes in the NBF and MF groups beyond 12 months. As the feeding categories related only to the first 6 months postpartum, analysis of weight change beyond 12 months would not be meaningful.

The independent variables assessed included maternal HIV status, antenatal CD4^+^ count (≤200, 201–349, ≥350 cells/mm^3^) and viral load (<100 000, ≥100 000 copies/ml) in HIV-infected women, socio-demographics (highest level of education, current school attendance, mother main income provider, water source, toilet type), pregnancy-related (parity, gestation age at booking based on the first day of the last normal period, number of multiple births), infant characteristics (sex, birth weight), and feeding modality as prior studies have examined the associations between these variables and postpartum weight (Ladner *et al*. 1998; Papathakis *et al*. 2006; Murnane *et al*. 2010; Koyanagi *et al*. 2011).

A generalised estimating equation (GEE) mixed model with an autoregressive correlation structure was used to assess mean weight changes from the first visit by maternal HIV status and control for confounding factors. Single imputation was used to estimate missing weights with the last known value substituting for missing data. The mixed model analyses weight from first to last visit accounting for mortality and loss to follow-up. Univariable analysis was conducted for main effects by HIV status. Variables significant at *P *<* *0.2 univariably were tested in multivariable model; variables significant at *P *<* *0.05 were retained. Time (months from delivery) was significant in all models. Separate models were fitted to determine the mean weight change in HIV-infected and -uninfected women, adjusted for baseline weight. A model was also fitted to determine the mean weight change in HIV-infected relative to -uninfected women at each time point from baseline to 24 months. Slopes for mean weight change in HIV-infected and -uninfected women may differ over time postpartum (Koyanagi *et al*. 2011). Hence, an interaction term for time and HIV status was included in the multivariable model to determine factors associated with mean weight change. Multivariable models were adjusted for maternal age, other socio-demographic factors and baseline weight (there was a significant difference in baseline weight between the groups and time since delivery).

Separate models were fitted for the following: (i) HIV-infected women to examine the effect of antenatal CD4^+^ count and viral load on weight change and (ii) HIV-infected and -uninfected women for each 5 month feeding modality (restricted to 12 months due to small sample size in the NBF and MF groups beyond 12 months). All analyses were conducted using SAS 9.2 (SAS Institute Inc., Cary, North Carolina, USA).

## Results

Of 2875 women with live-born infants in the VTS, 2340 were included (Figure 1). Compared to included women, those excluded had similar baseline weights (*P *=* *0.25), shorter median EBF duration (*P *<* *0.0001) and were less likely to be main income providers (*P *<* *0.0001), or own refrigerators or freezers (*P *<* *0.0001). Follow-up duration was not different between those included and excluded (*P *=* *0.07) (data not shown).

Median duration of EBF was 179 days (IQR: 149–180 days) for HIV-uninfected women and 175 days (IQR: 83–180 days) for HIV-infected women (data not shown). Significantly more HIV-infected than -uninfected women were reported to be main income providers (Table 1). HIV-infected and -uninfected women did not differ in access to piped water and highest level of education (data not shown). HIV-infected women had median antenatal CD4^+^ count of 450 cells/mm^3^ (IQR: 312–627 cells/mm^3^) and antenatal viral load of 12 000 copies/ml (IQR: 3600–42 000 copies/ml). Only 10.9% of HIV-infected women had advanced immunosuppression (antenatal CD4^+^ count <200 cells/mm^3^) (data not shown).

**Table 1:** Baseline characteristics of 2340 women in the VTS study by HIV status*

Characteristic	HIV-uninfected	HIV-infected	*P*-value
Median maternal age, years	21.8 (19.1–28.4)	25.1 (21.5–29.6)	<0.0001
Median baseline weight, kg	62.6 (56.3–70.8)	62.1 (55.9–69.6)	0.08
Median time from baseline to last visit, weeks	56.1 (32.0–88.1)	52.7 (27.4–83.9)	0.01
Antenatal clinic enrolled			
Rural	669 (55.9)	506 (44.3)	<0.0001
Periurban	353 (29.5)	411 (35.9)	
Urban	175 (14.6)	226 (19.8)	
Mother main income provider			
Yes	73 (6.1)	132 (11.5)	<0.0001
No	1116 (93.2)	991 (86.7)	
Missing	8 (0.7)	20 (1.8)	
Currently schooling			
No	1088 (90.9)	1089 (95.3)	<0.0001
Yes	103 (8.6)	43 (3.8)	
Missing	6 (0.5)	11 (0.9)	
Type of toilet†			
None	300 (25.1)	251 (22.0)	0.03
Non-flush	708 (59.1)	665 (58.2)	
Flush	183 (15.3)	215 (18.8)	
Missing	6 (0.5)	12 (1.0)	
Water source			
River, stream, dam, pond	272 (22.7)	247 (21.6)	0.30
Borehole, well, tank	93 (7.8)	92 (8.1)	
Piped	825 (68.9)	789 (69.0)	
Missing	7 (0.6)	15 (1.3)	
Gestation at booking, weeks			
0–12	22 (1.8)	28 (2.5)	0.0028
13–28	792 (66.2)	671 (58.7)	
>28	377 (31.5)	438 (38.3)	
Missing	6 (0.5)	6 (0.5)	
Parity			
0	591 (49.4)	379 (33.16‡)	<0.0001
1–3	468 (39.1)	673 (58.88)	
4–11	126 (10.5)	83 (7.26)	
Missing	12 (1.0)	8 (0.7)	
Delivery setting			
Outside health services	182 (15.2)	183 (16.0)	0.0061
Clinics	325 (27.2)	377 (33.0)	
Hospitals	686 (57.3)	577 (50.5)	
Missing	4 (0.3)	6 (0.5)	
Mode of delivery			
Vaginal	1049 (87.6)	987 (86.4)	0.48
Caesarean section	146 (12.2)	152 (13.3)	
Unknown	2 (0.2)	4 (0.3)	
Infant sex			
Female	593 (49.5)	571 (49.96)	0.58
Male	604 (50.5)	571 (49.96)	
Unknown	0 (0)	1 (0.8)	
Infant birth weight, kg			
<2.5	86 (7.2)	118 (10.3)	0.01
≥2.5	1064 (88.9)	971 (85.0)	
Missing	47 (3.9)	54 (4.7)	
Number of infants			
Singleton	1176 (98.2)	1114 (97.5)	0.19
Twins	21 (1.8)	29 (2.5)	
Infant follow-up			
Infant death	32 (12.6)	141 (36.2)	<0.0001
Lost to follow-up, reason unknown	77 (30.3)	87 (22.3)	
Moved post-natally	145 (57.1)	162 (41.5)	
Maternal follow-up			
Died post-natally§	9 (3.9)	52 (17.7)	<0.0001
Lost to follow-up, reason unknown¶	75 (32.8)	87 (29.6)	
Moved post-natally	145 (63.3)	155 (52.7)	
Infant feeding modality up to 5 months			
EBF	920 (76.9)	762 (66.7)	<0.0001
MF	143 (11.9)	101 (8.8)	
NBF	125 (10.4)	275 (24.1)	
Missing	9 (0.8)	5 (0.4)	

EBF, exclusive breastfeeding; MF, mixed feeding; NBF, non-breastfeeding.

* Values are medians (IQR) or absolute numbers (percentage) as appropriate, *P* values are based on chi-square test or Fisher's exact test for categorical variables or Kruskal–Wallis test for medians, *n *=* *1143 (HIV-infected) and *n *=* *1197 (HIV-uninfected).

† Non-flush toilets included ventilated pit latrines, open pit latrines, chemical toilets or buckets.

‡ Data were not rounded up as percentages were adding up to more than 100%.

§ In the overall sample, 0.8% (*n *=* *9) of HIV-uninfected women and 4.5% of HIV-infected women died postnatally.

¶ In the overall sample, 6.3% (*n *=* *75) of HIV-uninfected women and 7.6% (*n *=* *87) of HIV-infected women were lost to follow-up post-natally.

### Postpartum weight change

Baseline median weight did not differ between HIV-infected and -uninfected women. Median baseline visit was at 6 weeks postpartum (*n *=* *1049); 281 women overall had a visit before 6 weeks: five at delivery, 29 at week one, 27 at week two, 14 at week three, 29 at week four and 177 at week five (63%). In 1356 women with heights, median baseline weight was 62.3 kg (IQR: 56.2–70.4 kg) and baseline BMI was 24.7 kg/m^2^ (IQR: 22.6–27.8 kg/m^2^). Median baseline weight in women without heights was 62.4 kg (IQR: 55.9–69.6 kg). Median baseline MUAC and the prevalence of low baseline MUAC (<23 cm) in HIV-infected and -uninfected women did not differ (data not shown).

**Figure 2:**
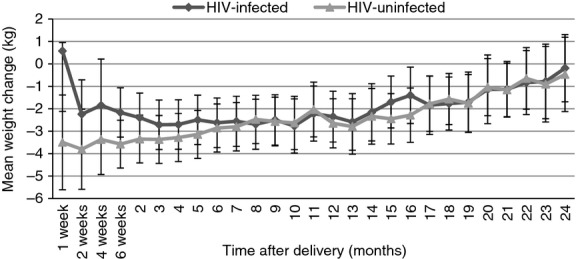
Mean postpartum weight change in HIV-infected and -uninfected women relative to baseline weight from delivery to 24 months. Line graphs represent the estimated change in weight (kg) from the baseline weight, across all the visits from delivery until 24 months, *n *=* *1143 (HIV-infected) and *n *=* *1197 (HIV-uninfected).

The weight change pattern was similar in HIV-infected and -uninfected women through 24 months (Figure 2). HIV-infected and -uninfected women lost approximately 2.0–3.0 kg between 6 and 13 months, then gradually gained weight through 24 months. At 24 months relative to the baseline weight soon after delivery, HIV-infected (−0.2 kg CI: −1.7 to 1.3 kg; *P *=* *0.81) and -uninfected women (−0.5 kg; 95% CI: −2.1 to 1.2 kg; *P *=* *0.58) had marginal and non-significant weight loss.

### Postpartum weight change in HIV-infected women by birth-to-5-months feeding modality

**Figure 3:**
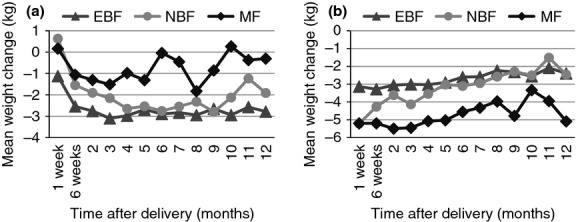
Mean weight change postpartum in HIV-infected (a) and -uninfected (b) women by feeding modality up to 5 months relative to baseline weight. Line graphs represent the estimated change in weight (kg) from the baseline weight, across all the visits from delivery until 12 months.

There were 990 EBF, 195 NBF and 119 MF still present in the study at 12 months. Mean weight loss from baseline to 12 months among 762 HIV-infected women who EBF at 5 months was 2.8 kg; mean weight losses from baseline to 12 months among 5 month NBF and MF were 1.9 and 0.3 kg, respectively. The mean weight loss of HIV-infected 5-month EBF women was similar and significant at each time point through 12 months relative to the baseline weight (Figure 3). Women in the 5-month NBF group had an estimated maximal weight loss of 2.8 kg at 9 months, decreasing thereafter. Five-month MF women had non-significant weight change postpartum from baseline through 12 months (Figure 3).

### Postpartum weight change in HIV-uninfected women by birth-to-5-months feeding modality

Among 920 HIV-uninfected 5-month EBF, the mean weight loss was 2.4 kg from baseline to 12 months; mean weight losses from baseline to 12 months among 5-month NBF were 2.5 kg and among 5-month MF was 5.1 kg.

The mean weight loss of HIV-uninfected EBF women was similar through 5 months with weight gain thereafter. Five-month NBF lost more weight in the early postpartum period up to 3 months than EBF. MF had significant weight change from baseline until 12 months, losing 4.6 kg at 6 months postpartum. The weight loss in HIV-uninfected women in the first 6 months (Figure 2) appears to be driven by early postpartum weight loss in HIV-uninfected 5-month NBF and MF (Figure 3).

### Factors associated with postpartum weight change in HIV-infected and -uninfected women

In the multivariable model adjusting for maternal age, HIV, timing of postpartum visit and baseline weight, HIV infection was associated with non-significant 0.2 kg weight loss by 24 months (Table 2). Every kilogram higher baseline weight was associated with 1.0 kg mean weight gain (95% CI: 1.02–1.04 kg; *P *<* *0.0001) (Table 2). Infant feeding practice was not significantly associated with mean weight change. Multiparity was associated with mean weight gain relative to primiparity. Mothers who were not the main income providers gained more weight than those without an external source of income; other socio-demographic factors were not significant.

**Table 2:** Factors associated with postpartum weight change in HIV-infected and -uninfected women in the VTS study

Effect	*n* (%)	β (95% CI)*	*P-*value
Baseline weight	2340	1.03 (1.02 to 1.05)	<0.0001
HIV Status			
HIV-infected	1143 (47.0)	−0.2 (−0.5 to 0.2)	0.30
HIV-uninfected	1197 (51.2)	0 (ref)	–
Infant feeding modality up to 5 months			
Never breastfeeding	400 (17.1)	0.03 (−2.5 to 2.5)	0.90
Mixed feeding	244 (10.4)	0.1 (−3.0 to 3.2)	0.78
Exclusive breastfeeding	1682 (71.9)	0 (ref)	–
Infant birth weight, kg			
≥2.5	2035 (90.0)	0.2 (−0.3 to 0.7)	0.39
<2.5	204 (8.7)	0 (ref)	–
Gestation at booking, weeks			
13–28	1463 (62.5)	0.4 (−0.5 to 1.4)	0.38
>28	815 (34.8)	0.4 (−0.6 to 1.3)	0.48
≤12	50 (2.1)	0 (ref)	–
Parity			
1–3 live births	1141 (48.8)	0.8 (0.5 to 1.1)	<0.0001
>3 live births	209 (8.9)	1.0 (0.3 to 1.6)	0.0026
0 live births	970 (41.5)	0 (ref)	–
Currently attending school			
Yes	146 (6.2)	0.3 (−0.2 to 0.9)	0.26
No	2177 (93.0)	0 (ref)	–
Mother main income provider			
No	2107 (90.0)	0.5 (0.0 to 1.0)	0.05
Yes	205 (8.8)	0 (ref)	–
Water source			
Borehole	185 (7.9)	0.1 (−0.4 to 0.7)	0.72
Piped	1614 (69.0)	0.1 (−0.3 to 0.5)	0.53
River	519 (22.2)	0 (ref)	–
Toilet			
Non-flush	1373 (58.7)	0.1 (−0.3 to 0.4)	0.62
Flush	398 (17.0)	0.2 (−0.3 to 0.7)	0.42
None	551 (23.5)	0 (ref)	–
Mode of delivery			
Caesarean section	298 (12.7)	0.7 (−1.9 to 3.3)	0.19
Vaginal delivery	2036 (87.0)	0 (ref)	–
Infant sex			
Male	1175 (50.2)	−0.3 (−0.6 to −0.02)	0.04
Female	1164 (49.7)	0 (ref)	–

* Data presented are beta coefficients, *n *=* *2340, which represent the estimated change in weight (kg) for each unit change in the effect, across all the visits from delivery until 24 months.

HIV-infected women with better immunological and virologic status antenatally had significant weight gain postpartum: women with CD4^+^ cell count ≥350 cells/mm^3^ had 1.1 kg greater weight gain (95 % CI: 0.4–1.7 kg; *P *=* *0.0013) than women with CD4^+^ cell count ≤200 cells/mm^3^ adjusted for baseline weight, feeding and time postpartum. Women with antenatal viral loads <100 000 copies/ml had 0.7 kg mean weight gain, compared to those with viral loads ≥100 000 copies/ml (95%: 0.1–1.4 kg; *P *=* *0.0197).

## Discussion

In this study of breastfeeding ART-naïve women, HIV-infected women had less weight loss in the first 6 months and at 12 months postpartum than HIV-uninfected women. However, this 6-month weight difference between the groups was temporary. Both groups experienced similar marginal weight loss by 24 months and weight change by 24 months in 5-month NBF, and MF was not significantly different from that in women EBF for 5 months after adjusting for socio-demographic, pregnancy and infant-related factors.

Studies assessing postpartum weight change in HIV-infected women are sparse; varying classifications of feeding practices and time period further complicate the interpretation of results. Three studies assessing weight change up to 6 months postpartum in lactating women found weight loss (Papathakis *et al*. 2006; Koyanagi *et al*. 2011) or less weight gain (Ladner *et al*. 1998) in HIV-infected women compared to -uninfected women. In our study, weight loss in the first 6 months postpartum was greater in HIV-uninfected than in HIV-infected women, but the weight change was similar by 24 months. Initial findings from the VTS showed significant weight loss by week 24 among HIV-infected breastfeeding women and weight gain among HIV-uninfected breastfeeding women (Papathakis *et al*. 2006). However, after inclusion of weight measurements over 24 months, stratified by feeding modality, we observe no difference in weight by HIV or breastfeeding practice at 24 months.

We expected that HIV-infected women, particularly those EBF, would have lower postpartum weight gain compared to -uninfected women. The effect of chronic HIV stress may trigger lactation energy sparing, including basal metabolic rate changes, caloric intake, physical activity or tissue store mobilisation. In our study, it is unlikely that women could have reduced their physical workload despite illness and demands of lactation. Regarding tissue store mobilisation, in a VTS substudy of 142 women, there were no differences between HIV- infected and -uninfected in fat or fat-free mass through 24 weeks (Papathakis *et al*. 2006). Postpartum weight gain has been noted among women in Gambia, Egypt, Taiwan (Butte & Hopkinson 1998) and Rwanda (Ladner *et al*. 1998). In Zambia, greater weight gain in HIV-infected breastfeeding women with low BMI relative to women with higher BMI, irrespective of breastfeeding duration (Murnane *et al*. 2010), was suggested to be an energy-sparing adaptation in undernourished lactating women (Murnane *et al*. 2010).

Postpartum weight changes have been noted among HIV-infected populations by feeding modality and duration. In Kenya, HIV-infected breastfeeding women experienced more weight loss than formula-feeders from the earliest post-natal visit through 5–9 months (Nduati *et al*. 2001). In Zambia, more women gained than lost weight between four and 24 months postpartum, but longer-duration breastfeeding was associated with less weight gain (Murnane *et al*. 2010). The initial mean weight loss in our study in all women is consistent with early postpartum weight loss due to intensity of breastfeeding. An unexpected finding was greater weight loss in the first 6 months in HIV-uninfected (driven by 5-month NBF and MF) than HIV-infected women. HIV-Infected women who did not breastfeed had advanced HIV disease (CD4 < 200 cells/mm^3^) and better socio-economic status than EBF women (Coovadia *et al*. 2007). While no difference in mean weight change was found between groups after adjustment for socio-economics and feeding, women with additional income had more weight gain, similar to Zimbabwe (Koyanagi *et al*. 2011). When we controlled for infant feeding, immunocompetent HIV-infected women had greater weight gain relative to those with advanced disease at enrolment. These weight changes in the HIV groups in the first 6 months were transient, and both groups had similar weight changes through 24 months. The mean weight change pattern postpartum in 5-month MF is likely due to varied feeding with breast milk and formula introduced from an earlier age, preventing the initial mean weight loss associated with lactation (Butte & Hopkinson 1998). Among HIV-infected women, feeding practice was not associated with postpartum weight change after adjusting for antenatal viral load and CD4^+^ cell count.

The association between parity and weight gain was consistent with results from studies in the United States and Brazil (Wolf *et al*. 1997; Coitinho *et al*. 2001; Cohen *et al*. 2009). Data on gestational weight gain, pre-pregnancy weight, pre-delivery weight and dietary changes were not collected during our study (Butte & Hopkinson 1998). The magnitude of these residual confounding effects remains unknown. Women with higher baseline weights were more likely to gain weight postpartum, similar to other African studies where weight gain is desirable (Holdsworth *et al*. 2004) and associated with the absence of disease particularly in HIV-infected women (Bentley *et al*. 2005).

Strengths of our study included frequent measurements of feeding modality and maternal weight, through 24 months with low mortality and loss to follow-up. While VTS data were collected before ART access (Bland *et al*. 2010), which may modify the effect of HIV infection on postpartum weight, the inclusion of non-breastfeeding and HIV-uninfected women helps to establish the background risk of postpartum weight change. Hence, this study confirms the findings of studies such as the randomised trial in Zambia that breastfeeding does not appear to be associated with maternal weight loss in HIV-infected women (Murnane *et al*. 2010) and would therefore not be deleterious to their health. This is particularly relevant given the revised South African guideline providing all pregnant and lactating women with ART (Department of Health Republic of South Africa 2013) which is likely to improve maternal health (Becquet *et al*. 2010), further minimising the differences in postpartum weight change among HIV-infected and -uninfected women. This theory would be supported by weight gain observed in HIV-infected women with high CD4^+^ cell counts.

There were several limitations. Given the low prevalence of underweight and substantial proportion of overweight and obese women, and high median CD4^+^ cell count, this study may be generalizable only to well HIV-infected women in resource-limited settings. Second, there may have been non-differential misclassification between the feeding groups, with bias towards the null, although estimates of feeding practice by HIV status were similar to prior studies (Bland *et al*. 2008). Third, this study did not consider other contributory weight loss factors, including physical activity, malabsorption, and energy intake and expenditure associated with HIV or other illnesses. Serious morbidity events occurred in <10% (Coutsoudis *et al*. 2010). Mothers with multiple births were included; there were no effect of multiple births in the univariable analysis. In terms of follow-up, adherence among the feeding groups was similar. There were fewer women who were NBF and MF initially; relatively the follow-up over time is likely to be similar between the groups, but the absolute numbers in MF and NBF became too small for meaningful analyses.

In developing countries, breastfeeding beyond 12 months is common (Miotti *et al*. 1999; Fawzi *et al*. 2002; Read *et al*. 2004). Although vertical transmission of HIV is concerning, replacement feeding may not meet ‘acceptable, feasible, affordable, sustainable and safe’ criteria (Nduati *et al*. 2000; Fowler & Newell 2002; Coovadia *et al*. 2007). Breastfeeding is likely to remain the predominant practice of HIV-infected women in sub-Saharan Africa, and rapid adoption of new WHO 2013 ART recommendations (WHO 2013) in sub-Saharan Africa for pregnant and breastfeeding women will reduce transmission risk and improve maternal health. In this study, breastfeeding did not increase postpartum weight loss likely to be detrimental to HIV disease progression and therefore remains the best option for promoting maternal and infant health.
